# A Review on Polymer Nanocomposites and Their Effective Applications in Membranes and Adsorbents for Water Treatment and Gas Separation

**DOI:** 10.3390/membranes11020139

**Published:** 2021-02-16

**Authors:** Oluranti Agboola, Ojo Sunday Isaac Fayomi, Ayoola Ayodeji, Augustine Omoniyi Ayeni, Edith E. Alagbe, Samuel E. Sanni, Emmanuel E. Okoro, Lucey Moropeng, Rotimi Sadiku, Kehinde Williams Kupolati, Babalola Aisosa Oni

**Affiliations:** 1 Department of Chemical Engineering, Covenant University, Ota PMB 1023, Nigeria; ayodeji.ayoola@covenantuniversity.edu.ng (A.A.); augustine.ayeni@covenantuniversity.edu.ng (A.O.A.); edith.alagbe@covenantuniversity.edu.ng (E.E.A.); samuel.sanni@covenantuniversity.edu.ng (S.E.S.); 2 Department of Mechanical Engineering, Covenant University, Ota PMB 1023, Nigeria; ojo.fayomi@covenantuniversity.edu.ng; 3 Department of Petroleum Engineering, Covenant University, Ota PMB 1023, Nigeria; emeka.okoro@covenantuniversity.edu.ng; 4 Department of Chemical, Metallurgical and Materials Engineering, Tshwane University of Technology, Private Bag X680, Pretoria 0001, South Africa; moropengl@tut.ac.za (L.M.); sadikur@tut.ac.za (R.S.); 5 Department of Civil Engineering, Tshwane University of Technology, Private Bag X680, Pretoria 0001, South Africa; KupolatiWK@tut.ac.za; 6 Department of Chemical Engineering and Technology, China University of Petroleum, Beijing 102249, China; babalola.oni@covenantuniversity.edu.ng

**Keywords:** polymer nanocomposite, polymer nanocomposite membranes, wastewater treatment, gas separation, polymer nanocomposites adsorbent, functionalization

## Abstract

Globally, environmental challenges have been recognised as a matter of concern. Among these challenges are the reduced availability and quality of drinking water, and greenhouse gases that give rise to change in climate by entrapping heat, which result in respirational illness from smog and air pollution. Globally, the rate of demand for the use of freshwater has outgrown the rate of population increase; as the rapid growth in town and cities place a huge pressure on neighbouring water resources. Besides, the rapid growth in anthropogenic activities, such as the generation of energy and its conveyance, release carbon dioxide and other greenhouse gases, warming the planet. Polymer nanocomposite has played a significant role in finding solutions to current environmental problems. It has found interest due to its high potential for the reduction of gas emission, and elimination of pollutants, heavy metals, dyes, and oil in wastewater. The revolution of integrating developed novel nanomaterials such as nanoparticles, carbon nanotubes, nanofibers and activated carbon, in polymers, have instigated revitalizing and favourable inventive nanotechnologies for the treatment of wastewater and gas separation. This review discusses the effective employment of polymer nanocomposites for environmental utilizations. Polymer nanocomposite membranes for wastewater treatment and gas separation were reviewed together with their mechanisms. The use of polymer nanocomposites as an adsorbent for toxic metals ions removal and an adsorbent for dye removal were also discussed, together with the mechanism of the adsorption process. Patents in the utilization of innovative polymeric nanocomposite membranes for environmental utilizations were discussed.

## 1. Polymer Nanocomposites for Environmental Applications

**Figure 1:**
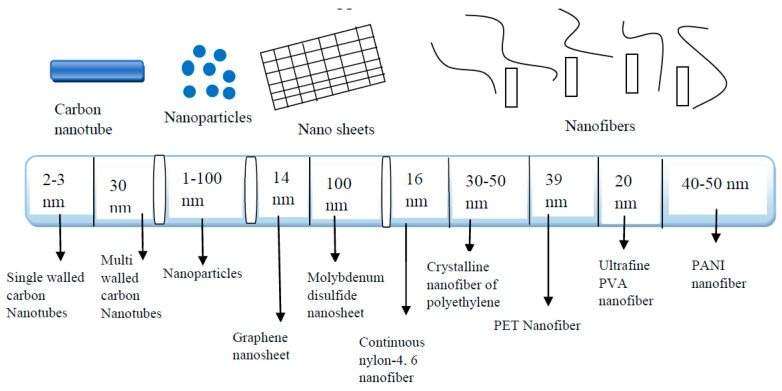
Schematic representation of different nanomaterials and their diameters. Carbon nanotubes are known to have diameters in the range of a nanometer. Nanoparticle properties change as it gets to nanoscale. The nanosheet is regarded as another type of nanoparticle. Nanofibers can be prepared from diverse polymers and hence, have diverse sizes.

Nanotechnology is the manipulation of materials at the infinitesimal or minuscule scale. The utilization of materials with different structures at the nanoscale spans in the range of 1 to 100 nm. Different types of nanomaterials have different diameter size (see Figure 1). The properties of nanomaterials, especially nanoparticles, exhibit some form of alteration as their sizes get close to the nanoscale; and as the segment of atoms at the surface of material gains momentum. As a result of its nanosized dimensions, nanomaterials such as nanoparticles; and carbon nanotubes, nanosheets, and nanofibers have demonstrated distinctive chemical and physical properties [1]. For example, they have huge surface areas to volume ratios or enormous interfacial reactivity. Table 1 gives the exceptional characteristics of different nanomaterials, which accord them the suitability for environmental utilizations when incorporated into polymers. For example, nanoparticles possess a huge surface area to volume ratio and high percentage of atoms/molecules associated with surfaces [2]. Carbon nanotubes possess a large length-to-diameter ratio (aspect ratio) that is higher than 1000 and a remarkable electrical conductivity together with a remarkable mechanical property [3]. What accords nanofibers with outstanding physical and chemical properties is their size, huge surface area, and high aspect ratio [4]. Nanosheets possess broad sideway dimensions and high surface area that makes them advantageous for the fabrication of excellent reinforced polymeric composites [5]. Furthermore, they have excellent catalytic activities such as photo-/thermo-catalytic activity [6]. 

In the sphere of science and engineering, studies on carbon nanotubes have expansively made them prospective materials, on the account of their enthralling properties such as high thermal and chemical stability, well-defined adsorption sites, and easy attachment of functional groups. On the account of structural defects that occur from the C=C bond breakages in the course of chemical treatment, the mechanical and electrical characteristics of single-walled carbon nanotubes can generally be transformed on functionalization. Hence, functionalization of the surface of carbon nanotubes is a vital element in ecological applications [7]. In the same vein, there is currently an establishment that the growth of nanoparticles has shown unambiguous interaction with contaminations in water and gases; this kind of properties spring up potential for the stimulation of innovative and improved environmental utilization of scientific knowledge [8]. However, the sizes of these materials at nanoscale carry matters related to mass transport and unnecessary drops in pressure when implemented in some suffuse systems [9]. Furthermore, some technical hitches in separation and recycle together with the imaginable threat to surroundings and human health are instigated via the possible emission of nanoparticles into the surroundings. However, a functional practice in curbing these technical hitches is to synthesize hybrid nanocomposite via the covering of fine particles over solid particles of larger size [9], which will be effective for wastewater treatment and gas separation. Regarding graphene-based nanomaterials for environmental applications, nanosheets represent a promising substitute 2D material that possesses several outstanding physicochemical and mechanical properties. These properties make them expressively different from those of graphene-based nanomaterials [10]. These attributes give them a prospective lead in new environmental phenomena and innovative utilizations. In the same vein, many nanofibers have demonstrated prospects for numerous emerging environmental applications [11]. Because they possess a very long length (which could be close to hundreds of kilometers) and are capable of being implanted within other media, they can be regarded as one of the safest nanomaterials for environmental applications.

Meeting the demand and effectually making safe freshwater available is one of the utmost challenges globally. It forms a foremost menace to safe utilization of water, well-being, and trade and industry development [25]. Additionally, the change in climate and pressure that comes from the economic advancement and industrialization have complicated the challenge of making adequate and safe drinking water available. The community and the industrialized sectors make use of large quantities of fresh water; in return, they produce enormous amounts of wastewater [26]. In the same vein, the upsurge in the concentrations of greenhouse gases such as carbon dioxide (CO_2_), nitrous oxide, ammonia, hydrogen sulphide (H_2_S), oxygen, and methane (CH_4_) in the atmosphere are causative for the transformation of global climate. Thus, the growing rate of air and water pollutions are certainly the known menaces to the environment, worldwide. Thus, consideration must be taken in finding lasting solutions to them.

The effort of industrial processes, such as the treatment of fumes from coal-fired plants, particularly for CH_4_ and CO_2_ removal in order to reduce their effects on greenhouse, should be intensified. In addition, wastewater treatment processes should be strengthened. Though there is growing interest in the application of diverse separation and purification technologies for removal of greenhouse gases and treatment of wastewater, investigation should be directed towards cutting-edge technologies in order to be able to create a clean environment [27]. In addition, the utilization of particles, even at the nanoscale size brings concerns relating to mass conveyance and extreme pressure drops when used in fixed beds or any suffuse devices. Again, challenges in separation and reuse together with potential hazards to the environment and well-being of the people are instigated by the possible discharge of nanoparticles into the surroundings. In order to overcome all the challenges involved with the use of nanoparticles, it is necessary to synthesize hybrid nanocomposites through the impregnation of fine particles over solid particles of huge size [28].

The fundamental reason for the uniqueness of the properties of polymer nanocomposites is their demonstration of distinctive physicochemical properties that are difficult to access when discrete components act alone. This is because of the large interfacial area that is present between polymers and nano-fillers. Hence, polymer composites are materials that have several phases in which reinforcing fillers are included within a polymer matrix; the resultant material has synergistic mechanical properties that is impossible to attain from just a component. Polymer nanocomposites combine advantages of both nanofillers and polymers due to their exceptional mechanical properties and compatibility, and distinctive physical and chemical properties. However, the decision to choose polymeric supports is generally governed by their thermal and mechanical characteristics. Supplementary property that should be considered is the choice of the organic hosts, which are the balance of hydrophobic/hydrophilic, bio-compatibility, chemical stability, electronic properties, and chemical functionalities [29]. The fabrication of polymer nanocomposites with the consideration of the above-listed properties offers a functional method that will overcome the problems of some nanofillers in environmental applications such as separation and reuse [30]. Environmental nanotechnology is known for its vital contribution in shaping present-day environmental science and engineering [30]. The mission of offering appropriate treatment provision for all contaminating sources is quite complex and costly. Hence, there is an augmented call for state-of-the-art and inexpensive technologies [31]. This report reviews the utilization of polymer nanocomposites for environmental applications such as membranes for wastewater treatment and gas separation, adsorbents for heavy metals, and dye removal. In addition, patents in the utilization of innovative polymer nanocomposites for environmental applications were also reviewed.

## 2. Polymer Nanocomposites Membranes

**Figure 2:**
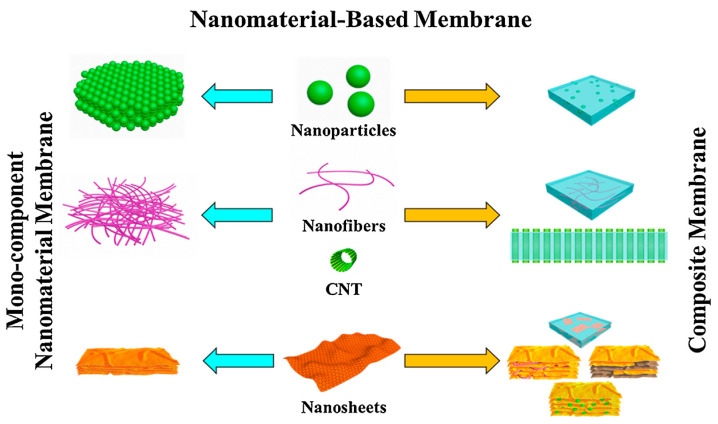
Membranes fabricated with nanomaterials, such as mono-component nanomaterial membranes fabricated with the integration of nanoparticles, nanofibers, carbon nanotubes, and nanosheets, and their composites (in conjunction with unique polymeric or inorganic materials) (Adapted from Ref. [37], with consent from Elsevier).

Membrane technology is attaining boundless significance in numerous industrial segments such as industrial effluent and wastewater treatment, food, medicine, pharmacy, biotechnology, and chemicals [32]. Presently, polymeric membrane is extensively been utilized for water treatment owing to its direct pore creating mechanism, smaller footprints needed for installation, higher flexibility, and moderately cost-effective when likened to inorganic membrane [33]. Nonetheless, polymeric membrane is restricted by some challenges, for example, the trade-off connection amid water permeability and solute selectivity together with low resistance to fouling [34]. Hence, the restrictions of present polymeric membranes provoked efforts in developing next-generation membranes that possess excellent penetrability and selectivity together with robust antifouling and chlorine resistance properties. The recent developments in nanotechnology afford an exceptional opportunity for membrane enhancement, because it is a permissive technology at the atomic level [35]. The use of nanomaterials in the synthesis of membrane has proven to be an excellent antifouling resistance and to perhaps, conquer the effect of trade-off amid the permeability of water and solute selectivity. Nanomaterials influence the selectivity, penetrability, mechanical strength, thermal stability, surface charge, hydrophilicity, and antibacterial characteristics of polymer membranes [36]. Hence, the contemporary developments in nanotechnology have huge potentials in membrane technologies, including densely packed nanoparticle integrated in polymers for the fabrication of membrane, and aligned nanotube membrane, nanofiber membrane, self-assembled two-dimensional stratum materials are their composites [37]. Figure 2 shows some collective membrane structures fabricated with nanomaterials. Furthermore, polymer nanocomposites have an important place in membrane materials because they enhance the transport properties of membranes and offer tremendous stability in an extensive span of operational conditions.

As a result of their potential to conquer the trade-off bond amid selectivity and penetrability, and the capability to mitigate membrane fouling problems in the course of water treatment applications, polymer nanocomposite membranes have gained considerable attention. They are considered as highly advanced membranes with high performance. Hence, polymer-matrix nanocomposite membranes are cutting-edge filtration materials with well-distributed nanomaterials in their polymer matrices. These membranes can be employed for liquid-liquid, gas-gas, and liquid-solid separations [34]. At the nanoscale level, the progress in polymer nanocomposites has stirred up interest—they have been utilized as novel and inexpensive techniques for membranes in water treatment and gas separation, adsorptive elimination, and discovery of waste product. Hence, the utilization of nanocomposite offers a prospective substitute for wastewater treatment, founded on the indication of the rising number of publications in the field, establishing increasing research interest [38].

### 2.1. Polymer Nanocomposites Membranes for the Treatment of Wastewater 

Membranes used for wastewater treatment are synthesized from many ranges of inorganic and organic materials [39]. Thus, nanocomposite membranes are a novel category of membranes made up of organic and inorganic polymeric materials, at nanoscale, thought to display improved performance when compared to typical membranes [40]. The inorganic materials comprise of ceramics, metals, and glass while organic materials comprise of polymers or mixed matrixes composite materials [41]. Recently, inorganic membrane synthesis has received consideration as a result of their excellent mechanical strength and chemical resistance; nonetheless, their relevance in water treatment applications is limited owing to the expensive synthesis process and snags in synthesizing [33]. Contrary to inorganic membranes, polymeric membranes are of better choice in industrial applications. The domination of polymeric membranes applications is due to their high selectivity, variation of membrane structures and characteristics, simplicity in preparation; control of the formation of pore and the polymers are not expensive [42]. Hence, the progression of membranes with excellent penetrability, excellent retention, and outstanding antifouling property is required for the decontamination of water [34]. 

The synthesis of nanocomposite membranes is for the preservation of the benefits of polymeric membranes. This is done in order to attain an exceedingly anticipated outcome for membrane advancement through the integration of engineered nanoscale materials in polymeric membranes. Furthermore, the drawbacks of polymeric membranes will be conquered [39,43]. Two approaches have generally been implemented to synthesize nanocomposite and thin film nanocomposite membranes. The first approach involves the deposition of engineered nanoscale materials on polymeric membrane surfaces. The second approach encompasses the synthesis of mixed matrix nanocomposite membranes via the straightforward frame of engineered nanoscale materials within the polymeric matrix [32]. An amalgamation of the two approaches has been studied [44]. Nonetheless, in order to avoid complex processes during the preparation of unique multifunctional nanocomposite membranes, coating/deposition and mixing can both be utilized to attain an extensive variety of membranes with various properties that could be tailored for anticipated use [39]. Deposition is the procedure of creating a stratum of engineered nanoscale materials on the dynamic surface stratum of a membrane in order to have control on the hydrophilicity of the surface of the membrane via the changing of the chemical groups that are seen at the surface [45]. Several engineered nanoscale materials are accessible for integration into polymeric membranes to form state-of-the-art solutions for water paucity by mitigating fouling, attaining excellent fluxes, and enhancing the chemical and physical properties of the membrane. Hence, several nanomaterials could be used as a filler of nanocomposite membranes. 

Carbon nanotubes have outstanding thermal, electrical, and mechanical properties [46]. They can change/modify the physico-chemical properties of a membrane [47]. The interior pores of carbon nanotubes behave as selective nano-pores; hence, carbon nanotube composite membranes exhibited an excellent penetrability that is devoid of a reduction in selectivity when equated to other kinds of membranes [48]. Dumée et al. described a strategy of preparing carbon nanotube composite membranes for the utilization of a direct contact membrane distillation by varying feed temperatures and operating conditions [49]. The membranes were also structurally characterized. The carbon nanotube composite membrane structures expressively revealed enhanced performance when likened to pure self-supporting carbon nanotube. The best carbon nanotube composite membranes attained higher permeabilities with 95% rejection of salt with a 39 h lifespan under continuous operation, which accords them prospective candidates for direct contact membrane distillation. Maphutha et al. synthesized a membrane from carbon nanotube-polymer composite using a polyvinyl alcohol barricade stratum for the separation of oil from wastewater [50]. The synthesis of carbon nanotubes was done via chemical vapour deposition with the employment of a phase inversion technique for mixing carbon nanotubes in a solution that is made of polymer composite, which was used for fabricating the membrane. The permeate demonstrates that concentrations of oil lower the conventional limits of 10 mgL^−1^, with an outstanding throughput and more than 95% rejection of oil was achieved [50]. Chan et al. synthesized a 1.5 nm diameter carbon nanotubes membrane with two zwitter ions at tip tails and attained ion retention of 100% [51]. Zhao et al. recently proposed an innovative polyphenol-metal influenced nanohybridization approach for the purpose of fabricating anti-oil-fouling carbon nanotube membranes to obtain excellent-flux, antifouling, and separation of self-cleaning oil/water [52]. The proposal was attained by taking into account facile synchronization arrangement and the strong metal ion embarkment competency of tannic-metal synchronization multiplexes. The polyphenol-metal influenced the nanohybridization approach, which falls within the arrangement of polyphenol-metal (chitosan/tannic acid-Fe^3+^) multifaceted primer on the carbon nanotube membranes through an in situ FeOOH nanorods mineralizating process. The carbon nanotube-chitosan/tannic-FeOOH nano-hybrid membrane attained excellent water permeability and notable high water recovery ratio [52].

In order to curtail fouling, diverse kinds of nanoparticles have been integrated into polymeric membrane matrices to reduce membrane hydrophobicity, membrane roughness, and improve surface properties [35]. Nanosilver (nAg) particles are effectual and are recognized as antibacterial agents. nAg could be integrated into membrane as a nano filler to neutralize microorganisms in the course of filtration [53], hence reducing membrane biofouling (Zhu et al., 2010). Nanosilver particles antimicrobial effects could be ascribed to their ability to interrupt the functions of cell membrane, disturb electron transport chains, and destroy cell protein and deoxyribonucleic acid [54]. Diverse oxidation states of silver (Ag^0^, Ag^+^, Ag^2+^) have been examined in biopolymer composite membranes [55]. The integration of nAg in membranes basically offer the capability of improving the properties of membrane anti-biofouling; however, they are usually not effective in enhancing the permeability and rejection ratio of the membrane [44]. Sile-Yuksel et al. investigated the influence of silver-nanoparticles locality in three different forms of polymer [56]. Three different polymers—polyethersulfone, polysulfone, and cellulose acetate—were utilized for synthetizing nanocomposite membranes at three diverse silver-nanoparticles ratios. A comprehensive account of how the location of silver-nanoparticles changes was reported in the study. The comprehensive account was dependent on the forms of polymer and the subsequent impact this locality has on the antibacterial properties of the membranes. It was reported that silver-nanoparticles are homogeneously positioned alongside the membrane matrix of both skin stratum and sub-stratum; however, they bulged from the top surfaces of polysulfone and polyethersulfone membranes. Accordingly, the upsurge of silver-nanoparticle/polymer ratio has a tendency to upsurge water permeability in polysulfone, whereas it was reduced by respectively employing polyethersulfone and cellulose acetate polymers. Haider et al. [57] presented the immobilization of silver-nanoparticles on polyethersulfone membranes through the introduction of amino groups, which aid the formation of aminated polyethersulfone (NH_2_- polysulfone, APES) [57]. The antibacterial activity was assessed alongside Escherichia coli. The annex of silver-nanoparticles on the surface of aminated polysulfone improved the bacterial/silver contact. Silver nanoparticles basically revealed a maximum disinfection ability through the reduction of the colony count of zero. Biswas and Bandyopadhyaya presented a report on the modification of the surface of polysulfone membranes by using silver-nanoparticles [58]. Polyethersulfone membranes were sulfonated by employing concentrated sulfuric acid for the generation of −SO_3_H groups on the membrane surface; it dissociates to provide SO^3−^ and H^+^ ions. Adding AgNO_3_ resulted in the replacement of H^+^ ions via Ag^+^ ions, and the silver-nanoparticles-polyethersulfone membranes demonstrated a constant permeate flow rate (3.45 Lh^−1^) as a result of total *Escherichia coli* cell-killing. Hence, the integration of silver-nanoparticles on membrane surfaces led to the improvement in antibacterial effect [58].

Titanium dioxide (TiO_2_) is a photocatalytic material with three diverse forms of crystals: anatase, rutile, and brookite. To a reasonably extent, TiO_2_ materials are not expensive when likened to the cost of other nanomaterials. TiO_2_ materials exhibit excellent thermal and chemical stability [59]. Furthermore, titanium dioxide, as a nano-material, enhances the penetrability, temperature resistance, and antifouling properties of membranes [60]. Ebert et al. found that polyvinylidene difluoride and poly (amide-imide) membranes combined with TiO_2_ as inorganic particle enhanced the resistance of temperature and permeability [61]. Madaeni et al. used polyacrylic acid and TiO_2_ nanoparticles to increase the antifouling property of polyvinylidene fluoride membrane [62]. The study shows that there was a notable improved fouling resistance on the account of the extraordinary yield resulted from and reduced TiO_2_ agglomeration [62]. Kim et al. [63] synthesized a hybrid composite membrane through the means of self-assembly of TiO_2_ nanoparticles. The membrane accorded substantial photo-bactericidal impact on Escherichia coli under ultraviolet light illumination. Vatanpour et al. [64] used three different types of TiO_2_ nanoparticles (P25, PC105, and PC500) with different sizes for the synthesis of mixed matrix polyethersulfone nanofiltration membranes [64]. The influence of forms and sizes of the nanoparticle on the structure, performance, and control of fouling was investigated. The higher the content of PC105 and PC500 TiO_2_ nanoparticles in the casting solution, the lesser the performance of the membrane, with P25 demonstrating better dispersibility; this is because of the agglomeration of these nanoparticles in polymer matrix. 

#### Mechanisms of Polymer Nanocomposites Membranes for Water Treatment

It is known that adsorption is the initial phase in the mechanism of water transportation and in other instances transportation of solutes through the membrane in the solution-diffusion model. This mechanism is affected by the membrane and charge of the molecules. This could boost the attraction or repulsion of forces between the membrane and molecules’ charges. Adsorption contributes to high retention; however, lower retention is usually noticed in the course of the gradual saturation of membrane [65]. Nonetheless, the removal mechanism of nanocomposite membranes in water treatment is size exclusion mechanism; it is the principal removal mechanism of metal ions, as the membrane composite functioned as a physical obstruction. Among the important elements that govern the mechanism of size exclusion are pores sizes and the sizes of the particles of the waste products under investigation [66,67]. Size exclusion model is an abridged retention model that is dependent on the physical size of a waste product. Generally, solutes larger than the membrane pore sizes are retained. Hence, size exclusion model was founded on the filtering phenomenon [68]. Several nanocomposite membranes used for water treatment contain pores at nanometer scale to attain selectivity. Usually, a small pore size that is less than 1 nm is used in order to make sure that high selectivity in the porous membrane via mechanism of size exclusion is attained [68]. Another mechanism found in the use of nanocomposite membranes for the treatment of wastewater is the electrostatic interaction (Donnan exclusion) amid the membrane and an external solution. For most nanocomposite membranes, the surface charge and filtering mechanisms have an impact on solute retention. These mechanisms are typically demonstrated by high rejection of divalent ions, low rejection of monovalent ions, and high flux [67]. 

### 2.2. Polymer Nanocomposites Membranes for Gas Separation

**Figure 3:**
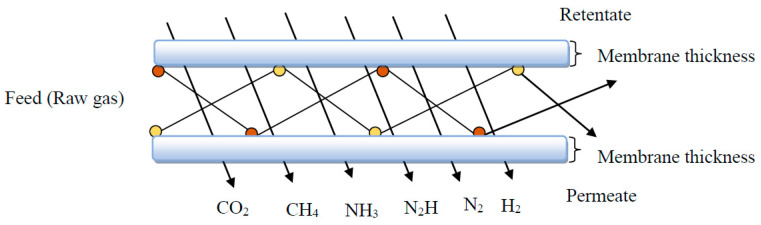
Schematic illustration of the membrane separation process using polymer membrane with selective inter-activities of the gas molecules with the pores across the membranes with the aid of Knudsen’s diffusion.

Over the past two decades, the emission of carbon dioxide has triggered many environmental complications. For the purpose of alleviating the concentration of carbon dioxide in the air, numerous approaches have been employed, and among them is the utilization of polymer nanocomposite membranes to successfully capture carbon dioxide [69]. Membrane separation process efficiently uses energy and it is a cost-effective tool in gas separation utilizations [70]. Presently, polymeric membranes are leading materials for gas separation processes, such as the recovery of landfill gas, sweetening of natural gas, flue gas and air separations, recovery of hydrogen, and purification. These materials possess the preferred mechanical characteristic and the tractability to be transformed into diverse modules [41]. Figure 3 depicts a straightforward representation of porous membrane gas separation, which uses Knudsen’s diffusion. The feed from the raw gas mixture is partitioned and moves into the permeate stream and retentate stream; however, this is contingent on the application. In preference, the membrane permits a constituent from the feed stream to move across the membrane in view of the selective interactivity of the gas molecules with the pores and sorption together with higher diffusivity of the same constituent due to variances in molecular size.

Nonetheless, the advancement in technology of polymeric membrane separation has been restricted on the account of a performance called upper bound trade-off curve amid gas penetrability and selectivity [71]. Owing to the performance of molecular sieve membranes such as zeolite, it is much above the upper bound trade-off curve, and scientists and scholars have pointed out that these materials are not easy to process and the cost of fabrication is high [72]. For the purpose of overcoming these restrictions, the interest in mixed matrix membranes has grown to materialization as a substituted method in membrane technology [73]. With regard to this method, superior gas separation features of molecular sieve materials with necessary mechanical characteristics and cost-effective processing capability of polymers are used [74]. A well-dispersed phase of inorganic materials in mixed matrix membranes possesses exceptional assembly and high mechanical strength. When these materials are integrated into the polymer matrix, there is an anticipation of the subsequent membrane characteristics having improved properties than the usual polymeric membranes [73]. Furthermore, the improvement in gas penetrability and selectivity by means of merging the fabrication of high-levels of performance membrane materials with present-day state-of-the-art membrane preparation technology is very essential for enlarging the industrial applications of polymeric membranes [75].

Scientists and scholars have investigated the fabrication of TiO_2_ nano-inorganic mixed matrix membranes in order to offer a solution to the polymeric membranes’ trade-off problem in gas separation [76,77]. Kiadehi et al. presented a report on the fabrication of amine functionalized TiO_2_ with TiO_2_ nanoparticles pretreated with ethelendiamine for gas separation [78]. The impacts of amin-functionalized TiO_2_ loading on the gas penetration of the mixed matrix membranes were examined through the variation of the amin- different functionalized TiO_2_ loading in the polysulfone matrix. Gas permeation measurements revealed that mixed matrix membranes implanted with a diverse quantity of amin-functionalized TiO_2_ revealed diverse separation performances. Owing to the enhanced interactivity of amine groups on functionalized-nano TiO_2_ with polar gases, amin-functionalized TiO_2_ offered excellent performances concerning penetrability and selectivity when likened to pure TiO_2_. Their study showed that the functionalization of TiO_2_ has boosted the gas penetrability of synthesized membranes [78]. Safaei et al. presented the fabrication and characterization of polystyrene-TiO_2_ nanoparticles mixed matrix membranes for the separation of CO_2_ from nitrogen. The impact of TiO_2_ nanoparticles loading was examined on membrane performance. Their study showed that upsurge of feed pressure led to reduction in flux of gases. There was a further confirmation that an excellent separation performance could be attained at TiO_2_ nanoparticles loading of 7 wt.% [79].

Carbon nanotubes are prospective functional materials that have recently gained much interest since swift movement of gases in carbon nanotubes was discovered because of low resistance movement in their intrinsic passages [80]. The exceptional features of carbon nanotubes inspire scholars to investigate an excellent penetrable, selective hybrid carbon molecular sieve membranes by embedding carbon nanotubes into membrane precursors [81]. However, the effectual employment of carbon nanotubes in mixed matrix membrane greatly depends on the capability to uniformly disperse the carbon nanotubes through the matrix. Rao et al. synthesized polyetherimide/multi-walled carbon nanotubes composite membrane for gas separation; carbon dioxide penetrability was roughly 27 × higher than polyetherimide carbon membrane, attaining 1463 Barrer, and O_2_/N_2_ perm-selectivity was 24.16 at 26 °C [82]. The results showed that the multi-walled carbon nanotubes offer an improvement in gas diffusivity by means of broadening the micro-pore volume. Tseng et al. [83] successfully fabricated multi-walled carbon nanotubes/carbon nanocomposite thin films by integrating multi-walled carbon nanotubes into polyimide precursor solutions [83]. The membrane displayed excellent CO_2_ flux of 866.6 Barrer, which was 2–4 × higher than that of pure carbon membrane. Sanip et al. [84] produced a mixed matrix membrane embedded with functionalized multi-walled nanotubes using the solution casting technique, whereby the functionalized multi-walled nanotubes were integrated into the polyimide membrane [84]. The influence of nominal multi-walled nanotubes content on the gas separation properties was studied. When likened to a neat polymer membrane, the mixed matrix membranes revealed 100% improvement in CO_2_/CH_4_ selectivity. The mixed matrix membrane possesses a high capability of separating gases at the molecular level. In addition, it possesses a high capability of ultimately reducing the energy consumed in modern-day separation processes. It was demonstrated from the study that the addition of carbon nanotubes to polymeric membranes has enhanced separation properties on the membranes to a definite extent. Sun et al. also fabricated mixed matrix membranes comprising of acid-treated functionalized multi-walled carbon nanotubes integrated into polyimide polymer for gas separation [85]. The gas separation performance revealed an increase of 292% CO_2_ permeability coefficient when 3 wt.% multi-walled carbon nanotubes was integrated into the mixed matrix membranes. Furthermore, there was an increase of 145% in the selectivity of CO_2_/N_2_ and an increase of 144% in the selectivity of CO_2_/CH_4_. Generally, carbon membranes attained from the thermal decomposition when polymeric membranes were utilized as precursors are projected as a prospective substitute for the separation of gas, primarily owing to their excellent separation ability when likened to polymeric membranes [86]. Fu et al. obtained carbon molecular sieve membranes by means of well-ordered pyrolysis of polyetherimide mixed with polyimide [87]. The results revealed that the free volume size and the pore size were improved with an upsurge in the polyimide content. Furthermore, there was a close connection amid the microstructures of polymer mixture precursors and carbon molecular sieve membranes. Their study established that the microstructure and gas separation performance of carbon molecular sieve membranes could be modified via the thermal blending of two stable polymers. Jiao et al. [88] used a poly [2, 2′-(p-oxydiphenylene)-5, 5′-bibenzimidazole] for pyrolysis under an inert Argon atmosphere for the production of alumina-supported carbon molecular sieve membranes utilized for CO_2_/CH_4_ gas separation [88]. There was an establishment of a structural performance relationship and it was deduced that high pyrolysis temperature could result in making a dense and well-organized graphite carbon structure and subsequently result in outstanding amalgamations of separation factor and gas penetrability of carbon molecular sieve membranes. 

#### Mechanisms of Gas Separation in Nanocomposite Membranes

The principal mechanism of the transportation of gas in some composite membranes is the Knudsen diffusion, while other composite membranes are principally modelled by sorption–diffusion or solution-diffusion mechanisms. The Knudsen diffusion mechanism is usually used to govern porous membranes. This mechanism makes use of viscous flow in narrow pores. The mean free path length of a gas molecule is strongly contingent on the type of the gas, the pressure, and the temperature [89]. Example of Knudsen diffusion mechanism occurs in Polyethersulfone incorporation with inorganic fillers of different shapes used for the separation of CO_2_/CH_4_. The study shows that the permeability of the membrane composites increased for both gases. The principal membrane mechanism responsible for gas transport is Knudsen diffusion; hence, CO_2_ and CH_4_ cannot really be separated [90].

Gas transport of dense solid materials is attained through the mechanism of solution-diffusion. For a considerably higher selectivity, this mechanism of transportation is subject to the solution and diffusion of the several components contained in the membrane phase. The mass transport in a solution-diffusion membrane comprises of three important steps [89]:

(1) Diverse components sorption from a feed in accordance with their partition coefficient amid the gas and polymer phase.
(2) The separate components diffusion contained in the membrane phase in accordance with their activity gradients.
(3) Components desorption from the membrane in the permeate gas phase.

Example of the solution–diffusion mechanism is the transportation of gas via carbon molecular sieve membranes. Gas molecules is specifically sorbed into the membrane at the upstream, which will then diffuse on the account of the influence of a chemical potential gradient, and lastly, desorb from the membrane at the downstream. Penetrability and selectivity are the two intrinsic properties, used in evaluating the performance of such membrane materials [91].

### 2.3. Working Principles of Membranes

Generally, the process of membrane separation is contingent on the existence of semi-permeable membranes. The general principle is relatively straightforward the membrane usually behaves as a precise filter that will allow the transportation of water to flow across the membrane. However, suspended solids and other matters are captured [92]. It is well known that the usual phenomena for reverse osmosis is that a semi-permeable membrane is positioned amid two partitions to permeate some species, and not permeate others. Furthermore, nanofiltration membranes are not a total barricade to dissolved salts. The principle of this filtration is contingent on the kind of salt and the kind of membrane, the salt permeability could be high or low [93]. Again, the principle of ultrafiltration membrane also involves the filtration of solutions or suspensions with the aid of pressure across a semipermeable membrane [94]. Both nanofiltration and ultrafiltration membranes possess pores that enable solvent and small molecules to be transported across the membranes and the larger molecules to be rejected. Hence, the mechanisms of nanocomposite membranes for water treatment and gas separation are based on the fact that the filtration principle is contingent on the type of solute and membrane, which could be influenced by the type of nano fillers integrated into the membrane. In addition, the mechanism is also contingent on the fact that membranes are occupied through a selective separation wall, where precise substances are allowed to move across the membrane while other substances are entrapped.

## 3. Polymer Nanocomposites as Adsorbent

Adsorption is a favourable and feasible process because of its low cost and high efficiency. Several types of carbonaceous materials have been employed as adsorbents for different adsorption processes on account of their exceptional performance for different process applications [95]. However, the advancement of exceptionally selective adsorbents for the elimination of toxic metal and organic contaminants are in demand, globally [96]. Polymer nanocomposite adsorbents have recently been seen as prospective materials for the elimination of different contaminants from wastewaters, with respect to sturdy mechanical strength, outstanding hydraulics performance, high stability, and turnable surface chemistry. Generally, the adsorption of the aimed contaminant greatly depends on the pore structure, physicochemical structure of the adsorbent material, surface function, and encapsulated moieties [97]. Nano adsorbents provide excellent sorption efficacy and swift process kinetics. This is on account of the large surface area they possess and easy accessibility of sorption sites [98]. Both material design and adsorption kinetics have systematically been studied [99]. Furthermore, the noteworthy adsorption performance, inexpensive, and extensive availability are responsible for the extent of the excellent attention it has gained [100]. In the following sub-section, the utilization of polymer nanocomposites as adsorbent for the elimination of metals ions, dye removal, and separation of gases is systematically reviewed.

### 3.1. Adsorbent from Nanocomposites Polymer for the Elimination of Metals Ions

Wastewaters from the industries comprising of metal ions are indirectly or directly released into streams, lakes, and rivers [101]. Usually, heavy metals cannot be decomposed or biodegraded; hence, they are stable environmental contaminants. In recent time, metal ion pollution in wastewater is globally the main ecological challenge that is dangerous to human life because of the mobility of contaminants in natural water bodies and their noxiousness [102]. Numerous approaches such as ion-exchange, membrane filtration, and electrochemical and adsorption technologies are employed for the elimination of several metal ions from wastewater [103]. Adsorption is most widely used among them as a result of low capital cost of the technology, simple strategy, ease of working principles, and insensitivity to toxic pollutants [104]. Furthermore, adsorption does not lead to the creation of destructive substances [105]. Factors such as solution pH, adsorbent dosage, contact time, concentration of initial metal ions, and temperature impact the adsorption of metal ions [106].

In the last decade, polymeric adsorbents have become apparent as prospective substitutes to conventional adsorbents with respect to their enormous surface area, modifiable surface chemistry, excellent mechanical stiffness, sizes of pores and distribution, and practicable reinforcement under minor conditions [107]. Conductive polymers consist of molecules, which are organic in nature, and have the capacity to conduct electricity and mimic metals through the possession of mobile electrons as charge carriers. Such polymers could display semi-conductivity. Generally, conductive polymers are in nature non-thermoplastic and have its place in the class of organic material [108]. Basically, there has been extensive study on conducting organic polymers such as polyaniline, polypyrrole, and polythiophene over the last two decades as a result of their excellent electrical conductivity, and good environmental stability [109]. These conductive polymer based-adsorbents and their composites have stimulated substantial consideration as a result of their possible utilization as adsorbents for metal ions such as iron, manganese, magnesium, arsenic, cobalt, nickel, etc., from wastewater [102,103].

Polyaniline and its derivatives have shown good adsorption ability because of their large quantity of amine and imine functional groups in polymer chains. Hence, they are employed as adsorbents to eliminate metal ions from wastewater. Researchers have recently investigated polyaniline and its derivatives as effectual nanoadsorbents for the elimination of metal ions [99]. Polyaniline particles have been assessed for the removal of mercury ions from aqueous solutions [110]. It was discovered from the study that the capacity of the adsorption of polyaniline particles was 600 mg g^−1^ for mercury ions [110]. Additionally, pH of 5.5 was suitable for the adsorption capacity of mercury ions owing to all the nitrogen-comprising functional groups on the polymer matrix, which might be responsible for mercury adsorption. Another metal ion of interest is chromium ion. There is an increasing attention on hexavalent chromium Cr(VI) due to its noxious and carcinogenic impacts on human health and its wide-ranging utilizations in industries like mining and electroplating [111]. Riahi Samani et al. presented a study on the adsorption of Cr(VI) ions in aqueous solutions by polyaniline particles [112]. These authors established that the kind of solvent utilized for the production of polyaniline had a distinct influence on the polyaniline morphology and its capacity to eliminate Cr(VI) from the aqueous solution. Furthermore, the elimination efficiency peak of Cr(VI) (more than 90%) was attained using the produced polyaniline. In accordance with these studies, it was finalized that the mechanism of polyaniline responsible for the removal was the amalgamation of surface adsorption. Samani and Borghei also presented a study on the absorptive characteristics of polyaniline prepared in 50/50 volume ratio of water and acetonitrile [113]. The prepared polyaniline powder was utilized as an adsorbent for the elimination of noxious hexavalent chromium from aqueous solutions. The optimal pH for the total removal of chromium was 7 (neutral condition) and pH of 3 (acidic condition) offered a maximum removal of hexavalent chromium. The maximum estimated adsorption of chromium was 36.1 mg/g for polyaniline [113].

Despite the successful use of polyaniline for adsorption, polyaniline is subjected to crucial shortcomings, which include inseparability, very low solubility when used in most obtainable solvents, shoddy conductivity when likened to metals, poor technology effectiveness, moisture absorbing, and unstable structure. With the purpose of improving the properties of polyaniline, different sorts of polyaniline composites have been synthesized with metals, metal oxides, diverse nanotubes, etc. A study has confirmed that polyaniline nanocomposites demonstrated value-added physical and physiochemical properties [108]. Scholars have employed polyaniline/polyaniline composites as adsorbents for inorganic and organic pollutants [114,115]. Wang et al. [116] prepared polyaniline nanoparticles and 1-D nanostructures by means of a facile sono-assisted chemical oxidation technique in protonic acids such as hydrochloric acid, surfulamic acid, citric acid, taurine, and deionized water for the elimination of aqueous Cr(VI) [116]. Their result revealed that all nanocomposite adsorbents demonstrated effectual adsorption for Cr(VI). Nonetheless, the extent of their adsorption efficacies is dependent on the protonic acids and the resultant molecular structures of polyaniline. In all, the polyaniline-hydrochloric acid synthesized in strong hydrochloric acid showcased maximum adsorption capacity, followed by polyaniline-surfulamic acid, polyaniline-citric acid, polyaniline-taurine, and polyaniline-deionized water. The capacity of the adsorption was equivalent with oxidation state and the extent of polyaniline protonation.

Polypyrrole adsorbents also have a substantial role in the elimination of metal ions owing to their simplicity and high flexibility in synthesis, high conductivity, biocompatibility, stability, good mechanical properties, and redox properties [117]. As much as adsorption efficiency is mostly influenced by the preparation conditions of polypyrrole, some attempts have been carried out to eliminate metal ions by means of polypyrrole conducting polymers made via chemical oxidative polymerization of pyrrole in the company of several oxidants at various conditions [109,118,119]. Zhang et al. [120] reported polypyrrole-based adsorbents synthesized by means of doping polypyrrole with chloride, polypyrrole with dodecyl sulfate, or polypyrrole with octadecyl sulfate and by aminating polypyrrole with aminopropyl-triethoxy-silane [120]. A huge quantity of bovine serum albumin was adsorbed on polypyrrole with chloride adsorbent at roughly pH of 5; however, nearly no lysozyme was adsorbed at the same pH. Contrastingly, a substantial quantity of lysozyme was adsorbed on polypyrrole with dodecyl sulfate or polypyrrole with octadecyl sulfate adsorbent at roughly pH of 10; however, nearly no bovine serum albumin was adsorbed by these two types of adsorbents at the same pH. Nonetheless, polypyrrole with aminopropyl-triethoxy-silane adsorbent exhibited a dissatisfactory selectivity for bovine serum albumin or lysozyme adsorption in the pH range of < 5 ≤ 9. Abdi et al. [121] presented a study on the adsorption of uranium (VI) ions on the polypyrrole adsorbent [121]. The investigation was done to examine the impact of a number of surfactants on the prepared polymers and their performance as the uranium adsorbent. The results revealed that the adsorption capacity peak of polypyrrole for uranium (VI) was determined to be 87.72 mg g^−1^.

As a result of polypyrrole facile and simplicity in synthesizing the adsorbent at low-cost, excellent electric conductivity, eco-friendly nature, and simplicity in modifying into nanocomposites has stretched its utilization away from the initial scope of conventional polymers. By putting these properties into consideration, the fusion of nanomaterials in polypyrrole for the fabrication of polypyrrole-based nanocomposites is presently getting a huge response as useful resources for strategically synthesizing highly-effective adsorbents to combat environmental challenges [122]. Functionalized magnetic nanoparticles of magnetite and maghemite have recently been employed for the elimination of metal ions, principally because of their stimulating features like large surface area, high saturation magnetization, and huge active sites numbers for metals adsorption. Furthermore, on account of their magnetic properties, they possess the ability to be separated from aqueous media without difficulty via the use of a proper magnetic field [123]. Especially when possessing very small size (within 10 nm), iron oxide particles are found as maghemite, which has a more stable phase, because it is likely to quickly oxidized [124]. Muliwa et al. [125] presented a report on the strategy and synthesis of a modest pilot-scale magnetic adsorption separation apparatus for the treatment of aqueous solution stream burdened with extremely noxious and transportable hexavalent chromium (Cr(VI)) ions by employing super-paramagnetic crystalline polypyrrole-magnetite nanocomposite as a mobile forager [125]. It was shown from the results that 100% Cr(VI) removal achievable from a continuously operated magnetic adsorption separation system and magnetic separation efficiency was 92%. Aigbe et al. [126] studied the impact of a fluctuating rotating magnetic field by employing a 2-pole three-phase induction motor for the elimination of hexavalent chromium ions from wastewater. This was done by employing a polypyrrole magnetic nanocomposite. The removal of hexavalent chromium ions was discovered to be dependent on pH subject to the effect of a rotating magnetic field, and as the percentage elimination of ions decreases with an upsurge in pH.

It is imperative to note that carefully-designed polymer-based magnetic nanocomposites should stimulate stability amid the extraction of pollutants from wastewater and the separation of the adsorbent material from the wet milieu. The attainment of stability is important in order to reuse and thus avoid their dissemination to the milieu. The segregation of these magnetic nanocomposites particles from aqueous solution might be attained via the utilization of systems of magnetic separations, which could be likened to other solid-liquid separation systems, as they are precise [125].

### 3.2. Polymer Nanocomposites as Adsorbent for Dye Removal

In the past three decades, the movement and dispersal of dyes in water have been extensively studied on the account of their toxic effects to human beings, plants, animals, and aquatic entities. Discharging dyes to the water source reduces the quality of water. Dyes, textile, cosmetics, rubber, paper, leather, foodstuff, and plastic industries utilize massive amount of synthetic dyes for the purpose of giving colour to their products; and they consume substantial volumes of water, which generates a large substantial quantity of coloured wastewater [127]. The colours in wastewater are classify by their high oxygen demand, inconsistent pH, imperishable, and dependability to several oxidizing agents. On the account of complex constructions and their pronounced struggle to dilapidation, several wastewater effluents have turned out to be a challenge for decolourization and demineralization [128]. In textile industries, close to 25% dye is discharged in the course of dyeing process and 2–21% is the discharge into several ecological constituents through a direct process. Aquatic life finds it difficult to get enough sunlight because of the colour of dyes [129]. The noxious effects of dyes have gotten to an extreme push on the account of their high half-life period. Devoid of any acceptable treatment process, dyes continue to linger in the milieu for an extended period. The extremely multifaceted structure and synthetic foundation of dyes makes their removal relatively problematic [129]. Furthermore, as a result of dyes’ chemical structure, they behave as resistant to numerous chemicals, oxidizing agents, and heat that are biologically non-degradable. Hence, it is not quite easy to decolorize the effluents as soon as they are released into the aquatic environment. From several available methods employed for eliminating noxious waste from wastewater, the most vital methods are ion exchange, reverse osmosis, precipitation, and adsorption. Amongst these methods, adsorption technique is the most versatile approach extensively used for the removal of dyes [130,131].

**Figure 4:**
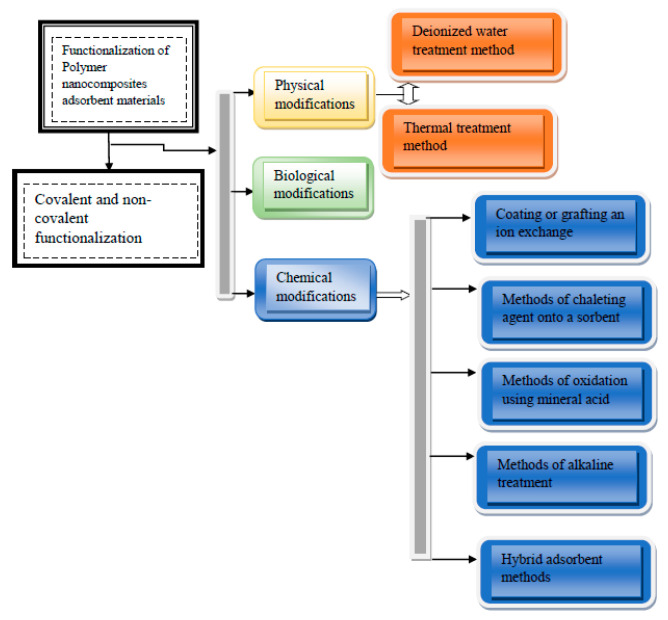
Classes of functionalization (covalent and non-covalent) approaches for adsorbent materials. Chemical modifications permit enhancement of their chemical and physical properties. The physical modifications are grouped into thermal treatment techniques and deionized water treatment techniques. Biological modifications involve the bioadsorbtion treatment method.

Currently, natural polymeric materials as adsorbents are attaining more interest for the treatment of wastewater due to their non-toxic and biodegradable nature [132]. Availability and low cost of polymer nanocomposites as adsorbent have encouraged their use for several commercial purposes, which includes the removal of dye from wastewater. Furthermore, polymer nanocomposites have also taken an important place when used as adsorbents because they possess excellent granulometric properties, a high surface area, boosted active sites, and are chemically and thermally stable. Functionalized polymer nanocomposites have captivated noteworthy attention on account of their chemical and physical features, simplicity of separation, and diverse reactive groups on the backbone chain [133]. In addition, due to the development in adsorbent synthesis and functionalization, there has been a solid prediction to ensure that adsorbents are prepared in an enhanced condition with an improved demonstration of the preferred qualities of an extremely competitive adsorbent [134]. This functionalization allows the adsorbents to be further responsive to binding with large dye molecules. Furthermore, it gives room for the behavior of adsorption and its subjection to a performance test in binary systems [135] and ternary dye systems [136]. Usually, raw adsorbent materials possess very low adsorption capacities; hence, the need to functionalize them preceding their utilizations [135,137]. There are different methods of functionalizing adsorbent materials; they could be grouped into three main classes (chemical, physical, and biological modifications), as shown in Figure 4. The mechanism of covalent functionalization comprises of the formation of covalent connection amid the carbon π-bond that is not saturated and other functional groups. In comparison to the noncovalent functionalization method, covalent bond functionalization is durable and sturdier [138]. Mainly noncovalent functionalization is dependent on π-π interactions, ionic interactions, and Van der Waals [138]. Functionalized adsorbent materials are however, tailored for different utilizations, such as adsorption of organic pollutant, metal ions, and dyes.

#### 3.2.1. Polymer Nanoparticle as Adsorbent for Dye Removal

There has been increasing attention on research in the advancement and applications of nanomaterials (nanoparticles, nanotubes, nanofibers, and nanowires) in science and engineering [139]. The cost and properties (particle size, shape, even distribution of particle size, structure of crystal, composition distribution, purity, aggregation control, and stabilization) of nanoparticles, made them appropriate for dye removal in wastewater. The large porosity of organic or inorganic nanoparticles and their small size and huge surface area take full advantage of their interface with the dye molecules via their several contaminant bindings sites, hence, their adsorption efficacy. From a kinetic perspective, the adsorption process facilitated via the nanoparticles is fast [140]. Hence, the utilizations of nanoparticles in the wastewater treatment process offer a novel channel of nanoparticles applications. Researchers have studied polymer nano adsorbents in water purification and dyes sorption using diverse kind of nanoparticles [141]. Nanoparticles such as iron oxides/hydroxides, zero-valent iron, iron oxide nanoparticles (such as magnetite, maghemite), titanium oxide, zinc oxide, and aluminium oxide nanoparticles together with their composites have been utilized as an adsorbent for the removal of dye from wastewater. In addition, there is consideration on the employment of polymer-based nanoparticles for the removal of dyes. Polymer-based nanoparticles possess abundant dormant usages like optical activity, increased catalytic, conductivity, toughness, and chemical selectivity [128]. However, nanoparticles of zero-valent metals, magnetic oxides, biopolymers, metallic oxides, and single-enzyme nanoparticles are usually employed for dye removal.

Magnetic nanoparticles have proven to be significant nano-adsorbents because they have the capability of easy separation. The adsorption process using this nanoparticle is done through the utilization of an external magnetic field, circumventing the monotonous and expensive separation technique of nano-adsorbents from aqueous solutions [142]. Furthermore, preferred functional groups can be hosted onto the surfaces of magnetic nanoparticles via several types of surface modification approaches, in order to channel the nanoparticles for precise utilizations [143,144]. From the investigation conducted by Zhang et al., [145], chitosan-coated octadecyl-functionalized magnetic nanoparticle was prepared for the elimination of perfluorinated compounds. The coating advances the dispersibility of magnetic nanoparticles in aqueous solution and improves the non-intrusion ability of the adsorbent to natural organic macromolecules in multifaceted samples. Atta et al. [146] directly prepared ultrafine well-dispersed Fe_3_O_4_-poly(acrylamide-co-sodium acrylate) core-shell magnetic nanogels in an aqueous solution using the controlled co-precipitation method. The composite was prepared for methylene blue removal from aqueous solutions. Their study revealed that for methylene blue removal, the adsorbents demonstrated high sorption capacities. Dai et al. [147] formulated a facile and eco-friendly route to synthesize magnetic nano-adsorbent for the elimination of pollutants from the environment. Founded on mussel-inspired polymerization, amino-coated magnetic nanoparticles were synthesized via immersion of magnetic nanoparticles into an aqueous solution of catechol and hexanediamine at a constant temperature, for the elimination of Congo red. The capacity of absorption for amino-coated magnetic nanoparticles was attained at 97.3 mg g^−1^ for Congo red, and the adsorption got to approximately 80% of the equilibrium adsorption quantity in less than 4 hours. Muntean et al. [148] developed a novel approach to selectively remove industrial dyes from wastewaters by utilizing the adsorption technique on the account of magnetic adsorbents. The effective removal of pollutants was dependent on initial pollutants concentration, temperature, magnetite/carbon nanocomposites dose, and solution pH, which upsurges with an upsurge in carbon content. Different optimum capacities of adsorption were attained for different dyes. Yadav et al. synthesized and characterized magnetic (Fe_3_O_4_)/activated charcoal (AC)/*β*-cyclodextrin (CD)/sodium alginate polymer composite gel beads for the adsorption of methylene blue [149]. Their results revealed that the rate of methylene blue removal was 99.53% in 90 min. The adsorbent obtained has unique features that allows a simple recovery devoid of any substantial weight loss of the adsorbent employed. This is the confirmation that sodium alginate-based material possesses excellent mechanical properties and optimum methylene blue adsorption capacity, outstanding restoration capability, and excellent separation properties.

Biopolymers are considered substances that are by nature formed through living species; hence, they are known to be ecologically pleasant and viable. Chitosan and cellulose are of precise importance on account of their ample availability, simple modification, and utilization prospective. The utilizations of biopolymer-based hydrogels and nanocomposite films are effectual biosorbents for complete removal of a collection of organic and inorganic contaminants, which includes xenobiotics from wastewater [150]. They have been specifically employed for the adsorption of metal ions and dye. Sadeghi-Kiakhani et al. [151] prepared a biopolymer chitosan-polypropylene imine as a biocompatible adsorbent for its reactive textile dyes (reactive black 5 and reactive red 198) removal potential. On account of optimum process parameters data used in the study, the performance dye removal of 97 and 99% were respectively attained for reactive black 5 and reactive red 198. As a result of high adsorption capacity of chitosan-polypropylene imine for reactive black 5 (6250 mg g^−1^) and reactive red 198 (5882.35 mg g^−1^), biocompatibility and eco-friendly properties of chitosan-polypropylene imine might be an appropriate adsorbent for reactive dyes removal from wastewater. Liu et al. prepared and characterized a nanoscale Davankov-type hyper-crosslinked-polymer as an adsorbent of benzene-ring-containing dyes and organic pollutants [152]. The hyper-crosslinked-polymer nanoparticles post-crosslinked from a poly(DVB-*co*-VBC) precursor prepared for the investigation were hydrophobic and stable. The as-synthesized hyper-crosslinked-polymer nanoparticles demonstrated a swift and selective adsorption capability to benzene-ring-containing dyes owing to its excellent conjugated structure. Kiani et al. synthesized magnetite nanospheres via the utilization of the solvo-thermal technique followed by modification of the surface by utilizing a synthetic polymer. The adsorbent was effectively employed for removing some anionic dyes from aqueous samples [153]. Saad et al. synthesized polyaniline nanoparticles for adsorption-based crystal violet removal via the ultrasonicated adsorption process [154]. The efficiency of the process was established based on the influence on certain conditions such as sonication time, temperature, dosage of adsorbent, and crystal violet concentrations. The process was validated via the estimation of various adsorption isotherms. The nanoparticles were efficient for 94.29% dye removal from wastewater.

Functionalized biopolymers (such as cellulose and chitosan) have recently attracted interests as emergent substitute approaches for organic dye removal. In addition, different monomeric units in biopolymers are connected through covalent bonds and functional groups, which relatively contributes to stable structure via cross-links amid network chains. For example, the ability of hydrogels to take up water and aqueous solution arises from hydrophilic functional groups linked to the polymeric backbone; however, their resistance to dissolution upsurges from cross-links sandwiched amid latticework chains [155]. Sharma et al. employed the functionality and properties of the surface area of graphitic-carbon nitride/zinc oxide incorporated in carboxymethyl cellulose for the adsorption of methyl violet from aqueous solution [156]. The existence of preferred functionalities on the surface of the nano adsorbent gave optimum interface-reaction with the molecules of methyl violet with optimum adsorption capacity of 96.43 mg/g. In a study by da Silver et al. [157], hydrogels of polyacrylamide grafted onto natural polysaccharides were made via standard and microwave-assisted techniques. The developed hydrogels were assessed as azo dye Acid Blue 113 adsorbent. The hydrogel offered the best properties to be utilized as an adsorbent of the AB 113 dye. The surface grafting of functional groups onto biopolymer surfaces has proven to be an effective modification procedure that boosted adsorption capacity [158]. Vafakish and Wilson [159] demonstrated the utilization of an adsorbent made from chitosan-based tweezers for fluorescein uptake with remarkable adsorption capacity. Their study contributed to the strategy of an exclusive category of improved chitosan materials through a facile synthetic method [159]. Hence, molecular tweezer-based adsorbents with the aid of a chitosan backbone for targeting aromatic guest species through adsorption can be developed.

#### 3.2.2. Adsorbent Made from Polymer Carbon Nanotube for Dye Removal

Several researchers have presented reports on improved adsorbents for the elimination of dyes from wastewater with the utilization of the modifications of carbon nanotubes with excellent functional groups. This type of functionalization method has resulted in a reduction in aggregation and hydrophobicity. This increases its capacity for adsorption, offers a very good distribution in aqueous solutions, and provides selectivity and affinity in the direction of the contaminants in the wastewater [160,161]. Carbon nanotubes probably comprise of functional groups, contingent on the preparation and purification process [7]. The functional groups of carbon nanotubes have made its usage possible with amalgamation with other materials to form composites for dye removal. Nonetheless, studies on polymer carbon nanotube composite, as adsorbent for the removal of dye is quite rare in comparison to polymer nanoparticle composite for the same purpose. Sheibani et al. [162] removed Congo red from aqueous solution using a multiwalled carbon nanotube oxidized and treated with HNO_3_. Excellent adsorption capacity of 357.14 mg g^−1^ was attained and it points to the efficiency and suitability of the nanotube for the attainment of a safe and clean environment. In a study by Abdi et al., [163], the zeolitic imidazolate framework was used as a metal-organic structure, based on graphene oxide, and carbon nanotubes; its hybrid nanocomposites were prepared by the facile approach at the surrounding temperature. The fabricated nanomaterials were employed as adsorbents for malachite green removal. The optimum adsorption capacities were 1667, 2034, and 3300 mg g^−1^ for zeolitic imidazolate framework, zeolitic imidazolate framework-carbon nanotubes and zeolitic imidazolate framework-graphene oxide, respectively, at 20 °C. The hybrid nanocomposites showed steady and extensive reusability of more than four cycles.

Although carbon nanotubes have been broadly employed as adsorbents for dye removal due to their small size, large surface areas, and exceptional chemical structure, the impact of unmodified carbon nanotubes on adsorption application is not quite satisfactory as a result of their agglomeration and being devoid of active adsorption sites [164]. Hence, the modification of carbon nanotubes that precedes the adsorption process is of significant interest owing to the efficiency of the modified composites. With the intention of enhancing the adsorption performance of carbon nanotubes for methylene blue removal, Zhang and Xu [165] improved carbon nanotubes wrapped with poly (sodium 4-styrene sulfonate) to attain carbon nanotubes/poly (sodium 4-styrenesulfonate). Their results proposed that carbon nanotubes wrapped with poly (sodium 4-styrenesulfonate) with the aid of π-π electron-donor–acceptor interactions and $-SO_{\bar{3}}$ groups can have the ability to accept the π -electrons from the conjugated system of carbon nanotubes. Hence, carbon nanotubes in the hybrid did not only adsorb methylene blue only via π-π interactions but also aided the electrostatic attraction amid $-SO_{\bar{3}}$ groups of poly (sodium 4-styrene sulfonate) and dimethylamino groups of methylene blue. Hosseinzadeh et al. [166] examined the prospect of utilizing improved multi-walled carbon nanotubes as an effectual adsorbent for several cationic dyes’ removal. The fabrication of the magnetic carbon nanotube composites were done by means of surface reversible addition fragmentation transfer of chain co-polymerization of acrylic acid and *N*-isopropyl acrylamide in the presence of magnetite nanoparticles. The composite adsorbent demonstrated high adsorption capacities. It is reusable and offers easy recovery, making it a good choice in the treatment of wastewater [166]. Gu et al. [167] employed polyethyleneimine-functionalized carboxylated multi-walled carbon nanotubes as an adsorbent for light green dye removal from solution. The adsorption of light green dyes solution attained equilibrium in 5 h of contact time with a removal of 97%. Yang et al. [168] reported a unique and straight forward biomimetic technique for Congo red removal. This was done by modifying the surface of carbon nanotubes with poly(ionic liquids). The data obtained revealed that the capacity of adsorption of the modified composite for Congo red removal was 50 mg L^−1^ higher than the capacity of adsorption of the carbon nanotube that was not modified. The modified composite for Congo red removal also possesses adsorption capacity of about 178 mg g^−1^ at pH of 7 [168].

Furthermore, the adsorption performance of some carbon nanotubes (such as pristine carbon nanotubes) is basically restricted owing to their poor dispersibility and without functional groups. Pristine nanotube possess some sidewall curvatures which are conjugated and exhibit an extremely hydrophobic surface; hence, the need for modification. Gan et al. [169] conducted their study on dopamine comprising of copolymers prepared via the ring-opening reaction amid dopamine and poly(styrene cooperate maleic anhydride) that were directly utilized for modifying carbon nanotubes with the aim of attaining functionalized carbon nanotubes composites (carbon nanotubes@poly (poly(styrene cooperate maleic anhydride) -dopamine)). Under several adsorption parameters, the functionalized (carbon nanotubes@poly (poly(styrene cooperate maleic anhydride) -dopamine)) composites adsorbents were employed for the removal of methylene blue. The results they obtained depicted that the capacity of adsorption of these modified carbon nanotubes towards methylene blue removal was close to four-fold when likened to pristine carbon nanotubes [169]. Mohammad-Salim [170] used pristine and oxygen functionalized multi-walled carbon nanotubes adsorbent to remove methyl orange dye. However, the results showed that the adsorption characteristics of the methyl orange dye are extremely affected by the medium pH. All the studies reviewed in this section have demonstrated that modified carbon nanotubes adsorbents with polymers aid the improvement of adsorption efficiency.

### 3.3. Polymer Nanocomposites as Adsorbent for Separation of Gases

Thin film processes have been utilized for reducing harmful gases from fossil fuel and natural gas. Organic matrix is the main thin film material used for reducing harmful gases because of its low-cost, mechanical properties, and permeability; however, selectivity restricts their desirable applications for industrial processes [171]. Hence, adsorption has become one of the prospective ways of solving the shortcomings of thin film composites [172]. The literature has shown that adsorbents synthesized from agrarian debris have been widely utilized owing to their outstanding performances and are inexpensive [173]. However, there is little information on polymer nanocomposites as adsorbent for separation of gases; nonetheless, there are other promising adsorbents for gaseous separation.

**Figure 5:**
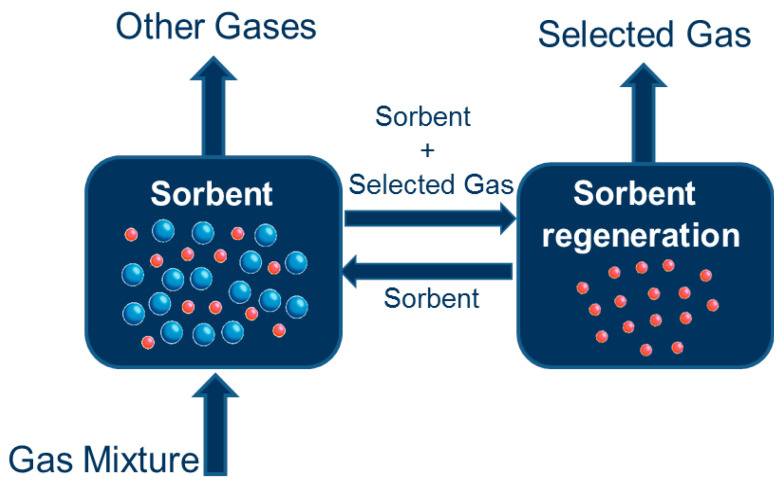
Illustration of the pressure swing adsorption technique. The technology works under pressure by using the adsorbent’s differences in gas adsorption rate to separate some gases from a gaseous mixture (Adapted from Ref. [174]).

The separation of gas using solvent/sorbents is dependent on gas affinity, in the direction of a precise sorbent like solid porous materials—activated carbon, zeolites, alumina, silica gels or a solvent, for example, methanolamine. The pressure swing adsorption method is the most illustrative technology. In this method, separation of gases ensues when the gaseous mixture makes contact with the sorbent/solvent, with further subjection to pressure. The gas that has maximum affinity for the adsorbent will be confined while the other gases move across the system (Figure 5). The restoration of the vessel is done by taking it back to air pressure or via upsurge in the temperature, by discharging the confined gas. The key benefits of this method are the high purity of the separated gas; however, a shortcoming is the high energy needed for running the system, particularly for the regeneration of the vessel [174]. Hence, for the continuous increase in the necessity for more effectual energy-saving, adsorbents that possess well channeled structures and tunable surface properties must be synthesized. Metal-organic frameworks made from metal-containing nodes linked by organic bridges are considered a new kind of porous materials that will meet the need for more efficient, energy-saving adsorbents for gas separation [175]. When evaluating metal-organic frameworks for gas adsorption and separation, the capacity of adsorption is a vital factor that should be considered [176]. Based on the large surface areas, amendable pore sizes, and satisfactory thermal stability they possess, they are prospective materials as adsorbents for gas separations.

Hu et al. [177] reported a microporous metal-organic structure with appropriate pore spaces favourably to absorb additional acetylene than ethylene whereas the functional amine groups on the pore surfaces put in force their inter-activities with acetylene molecules, further resulting in maximum performance. It was proposed that this material could be employed in industries for acetylene removal from ethylene/acetylene mixtures. Lin et al. [178] synthesized a quartz like metal-organic structure having a one-dimensional network for hydrocarbon separation. A substantial quantity of acetylene and the uptake capacities of CO_2_ and moderately high selectivities for the natural gases separation at surrounding condition were attained. Chen et al. [179] synthesized novel metal-organic framework-505@graphite oxide composites made of a copper-based metal-organic structure and graphite oxide through a solvothermal technique for effectual separation of CO_2_/CH_4_ and CO_2_/N_2_. The selectivities of these gases were assessed based on model adsorbed solution theory. Metal-organic framework-505@graphite oxide demonstrated a rise in upsurge of CO_2_ uptake of 3.94 mmol^-^g at 298 K and 100 kPa, possessing an upsurge of 37.3% when compared to the parent metal-organic framework-505. Chen at al. [180] presented an iron-based metal-organic structure PCN-250 for effective C_2_H_4_ decontamination from the mixture of C_2_H_6_/C_2_H_4_. There was a confirmation that C_2_H_6_ was adsorbed over C_2_H_4_ in PCN-250. In addition, PCN-250 possessed high C_2_H_6_ adsorption capacity, modest C_2_H_6_/C_2_H_4_ selectivity, and decent heat of adsorption. The adsorptive characteristic for the separation of C_2_H_6_ over C_2_H_4_ could be beneficial for entrapping C_2_H_6_ from C_2_H_6_/C_2_H_4_ mixtures, mainly imperative for entrapping low concentration C_2_H_6_ from the fractured gas mixtures in order to attain high-purity C_2_H_4_.

### 3.4. Mechanism of Adsorption

For any adsorption system, the mechanism of adsorption is of great significance. The adsorption phenomenon is contingent on inherent characteristics of adsorbents; hence, the fundamental mechanism is dependent on the characteristics of adsorbents. Therefore, it is important to examine the influence of several adsorbent characteristics on the quantity of uptake [181]. For a functional adsorption process, retention time and flow rate significantly contribute to the determination of inherent adsorption mechanisms. Azhar and Farshi [182] synthesized a starch-montmorillonite/polyaniline nanocomposite, which was then employed for a reactive dye adsorption. Their data showed that the mechanism of removal was dependent on the electrostatic attraction that occurs amid the molecules of dye and nanocomposite. For the polysaccharide-dye system studied by Wang et al., [183], innovative approaches such as molecular dynamic simulation, calculation from the first principles, and infrared (IR) analyses were used to determine the mechanism of Okra Polysaccharides–Methyl Violet interaction. The authors used adsorption isotherm models to investigate the mechanism of adsorption. The authors concluded that from the use of thermodynamic analysis, the adsorption was found to thermodynamically favour the exothermic process. The molecular dynamic study data, first-principles computation data, and the infrared analysis revealed that the mechanism of the process is pre-eminently electrostatic attraction between the adsorbent and Methyl Violet molecules [183]. In addition, it is important to note that diverse profiles for different isotherm curves could occur; this will affect the disparity in the adsorption process intrinsic mechanisms. This is because the feed in the adsorption process could comprise of several adsorbates; hence, a struggle of adsorption in the surface of the adsorbate will occur, and co-adsorption could subsequently ensue. A multi-component isotherm can therefore, be employed to illustrate this type of process [181].

## 4. Features of the Transport and Dissolution of Substances in Polymer Nanocomposites

The separation of polymer nanocomposite membranes is contingent on dissolution mechanism. Dissolution entails the conversion of polymer nanocomposites from their natural and particulate physical form into its ionic components [184]. Polymer dissolution into a solvent entails two transport routes, which are solvent diffusion and chain disentanglement [185]. Diffusivity is computed by measuring the mobility of discrete molecules that transient across the voids amid the polymeric chains in a membrane material [184]. The mechanism of dissolution comprises of the interactions of the infiltrating molecule at the molecular-scale in conjunction with the membrane polymer. The mechanism is contingent on the assumption that each molecule is sorbed at one interface of the membrane transported via diffusion through the membrane by means of voids amid the polymeric chains (or free volume), and desorbed at the other end of the interface [186]. This is attained by the affinity of the composite together with the polymer membrane, which is dependent on its capability to dissolve [187]. In addition, the mechanism is contingent on desorption, in which nano materials adsorbed at the polymer-liquid interface impulsively partition into the liquid [188]. During the course of diffusion and desorption, the nano materials released continue in nano particulate nature; however, their composition, morphology, or surface properties could change in the course of the release process or at the end of the release process. In dissolution, the nano materials do not remain in the particulate form at the end of the release process; nonetheless, subsequent reformation of nanoscale particles from the dissolved residuals is possible, should there be favorable conditions [188].

**Figure 6:**
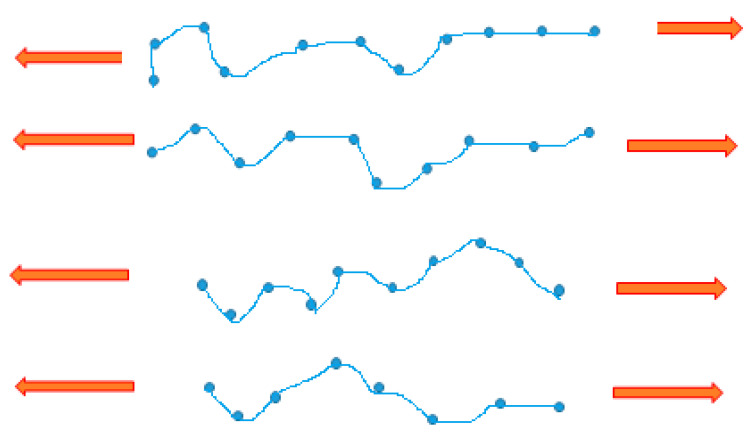
Rotation of the Kuhn segments resulting in alteration of polymer shape as a result of thermal vibration of atoms.

The transport operation of a given infiltrate is not the same for all polymers. The characteristics of nanocomposite transport is contingent on the free volume within the polymer and polymer chains’ segmental mobility [189]. Polymer molecules could be considered as chains that arise from flexible segments (known as Kuhn segments), entangled with one other. Their shape position can be changed through two means; which are rotation of the Kuhn segments and reptation of the whole molecule. The Kuhn segments in a polymer molecule can rotate in any direction owing to the thermal vibrations of the atoms (see Figure 6). As a result, the molecule can locally alter its shape within a blob or alter its whole shape under load; should there be any need for it. The rotations of the Kuhn segments results in polymer flexibility. Polymer chains segmental mobility is influenced by the degree of unsaturation, the degree of cross-linking the degree of crystallinity, and the type of substituent. Introducing bulky or polar substituents on a polymer chain affects the transport process. The transport properties is greatly influence by the glass transition temperature of polymers. Polymers that display low glass transition temperatures are accorded with a superior segmental mobility and higher diffusivity [190].

**Figure 7:**
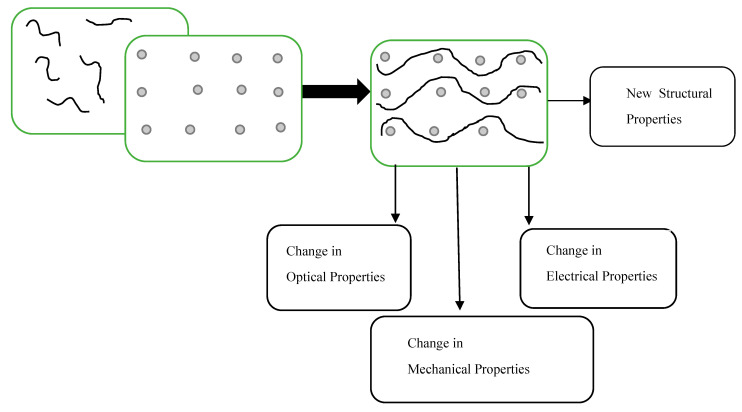
Illustration of the addition of nanoparticles into polymer matrices; it results in the alteration of optical, mechanical, and electrical properties in relation to the host polymer.

When nanoparticles are added into polymer matrices, the composite significantly alters the optical [191], mechanical [192], structural, and electrical [193] properties in relation to the host polymer. This is illustrated in Figure 7. Polymer-surface interactions is recognized to be instrumental in the determination of dynamics of interfacial polymers [194]; this governs the macroscopic characteristics of polymer nanocomposites [195]. Usually, attractive or repulsive polymer-surface interactions leads to an enhanced mobility or slower aging. This is documented in the study of thin films [196] or nanopores [197], for mobility in confined polymers. Furthermore, the inflexibility of the nearby polymer has a substantial impact on tuning the glass transition temperature (Tg) and dynamics of the confined polymer [195]. Chandran et al. [198] varied the glass transition temperature of polymer nanocomposite films driven by morphological transitions. The polymer nanocomposite films comprises of polymer-grafted nanoparticles embedded in a homopolymer matrix. Their study depicted a logical variation of the projected glass transition temperature T_g_, having a volume fraction of supplementary polymer grafted nanoparticles. The study interrelates the T_g_ variation under observation with the core morphological transitions of the nanoparticle dispersion in the films. A continuous upsurge in polymer molecular weight results in the decrease in the number of chain ends. The chain ends are characterized by a discontinuity that could create sites for permeate molecules to be sorbed into glassy polymers [199]. The mobility permeation in the polymer and the magnitude of sorption can be affected by the chain segmental mobility and the interface activities of different polymers. For an upsurge in molecular size from C_8_ and C_16_, the rate and magnitude of sorption decrease [189]. The transport phenomenon of the same polymer with the same cross-link density is contingent on the nature of the cross-link [189]. The integration of plasticizers to a polymer results in an upsurge in segmental mobility, such as the upsurge in penetrant transport.

## 5. Patents in the Utilization of Polymer Nanocomposites Innovations for Environmental Applications and Future Work

Discoveries in the progression of new polymer nanocomposites for environmental utilizations are highly inspiring and highlight promising technologies [25]. The advancement of polymer nanocomposites on the environment can be analyzed via intellectual property activities springing from the dynamic significance of securing innovations and technologies of interest [200]. A patent is an innovative idea that shows that, despite the fact that a new product could integrate some of the same technology as an existing product, there is still the possibility to acquire patent protection for new and inventive features. The modifications made to the existing technology might seem negligible when compared to the original invention; however, if such an invention offers a new benefit by operational law, patent protection could still be sought after. Hence, the significance of protecting an idea resulting from inventions and developments should not be undervalued [201]. Some inventions and patented products are discussed here for the purpose of having a greater understanding of the intrinsic environmental benefits of the utilization of polymer nanocomposites in environmental applications.

### 5.1. Patents on Polymer Nanocomposites Membranes for Environmental Applications

Rodrigues et al. [202] patented a filter that is modified with a polymer-carbon based nanomaterial nanocomposite envisioned to substantially enhance the performance of filtration and remediation of an extensive variety of chemicals, metal ions, organic matters, and living species. Polymeric materials, such as poly-*N*-vinyl carbazole, are joined with (1) graphene and/or graphene-like materials-based nanomaterials and (2) chemically modified graphene oxide with a chelating agent (ethylenediaminetetra-acetic acid). The nanocomposite was evenly deposited on the membrane surface. Lind et al. [203] patented membrane (100) for liquid separation. The membrane comprises of a polymer matrix (that is chlorine tolerant) that comprises of the thickness (115) and plurality of water-selectively-permeable particles (120), having a diameter that is approximately the same as the thickness of the polymer film, disposed within the said polymer matrix. The invention was aimed at molecular sieve inclusion nanocomposite membranes for liquid separations. Particularly, molecular sieve inclusion nanocomposite membranes possess high solute selectivity and have the capacity to withstand harsh chemicals and feed materials. Applications of molecular sieve inclusion nanocomposite membranes include osmotic processes (reverse and engineered osmosis) and pervaporation; though they are not limited to the listed applications. Lahalih [204] patented a nanocomposite mixed-matrix membrane. The membrane was synthesized from Halloysite nanotubes implanted in a hydrophobic polymer matrix. The nanotubes were a 1:1 layered alumino-silicate clay moulded into multi-layered hollow cylinders possessing walls, made from interchanging stratums of alumina and silica. The membrane was fabricated through the extrusion of the nanocomposite, which stretches over rollers in the course of the fabrication; the membrane was then annealed, cold stretched at ambient temperature, and then hot stretched. The subsequent membrane was microporous and could be employed as a membrane distillation. Bhattacharyya et al. [205] patented a nanocomposite membrane via the green synthesis route. The nanocomposite membrane comprises of a macroporous polymer membrane that exhibits a plurality of pores. A plurality of metal nanoparticles was synthesized and immobilized in the interior of the plurality of pores. The nanoparticles were abridged and covered with a green reducing and covering agent—green tea extract. The resulting nanoparticles were favourably protected from oxidation and agglomeration; and thus, offer more effectual and effective reductive degradation of 30 toxic chlorinated organic compounds and additional target contaminants of a water supply. There is also some patented work on polymer nanocomposites membranes for gas separation.

Kang et al. [206] patented a nanocomposite membrane that consists of an Ag-nanoparticle/polymer nanocomposite, which was evenly distributed in the polymer matrix, and a support membrane for aiding the nanocomposite, together with the process of synthesizing the membrane. The nanocomposite membrane is chemically stable, possesses an exceptional extended period of operation performance characteristics, together with exhibiting high selectivity and penetrability. Hence, it could be favourably employed for separating olefin from an olefin/paraffin blend. Fritsch and Merten [207] patented a composite membrane for separation of gases. The membrane possessed a selective separation stratum. The membrane is made of a producing polymer solution from at least one ammonium salt and at least one polymer, which is compatible with the ammonium salt. The polymer solution made a film that produces the selective separation stratum from the film via drying. An independent claim for the membrane is preferably for gas separation membrane. Vijayendran and Lalgudi [208] patented a polymer nanocomposite for the separation of an aimed gas from a second gas in a gaseous mixture. The composite comprises of (a) a matrix fabricated from a modified polymer, and (b) nanoparticles integrated in the matrix; the nanoparticles were functionalized for the purpose of having a stable interaction with the matrix. The improved polymer possesses a backbone that includes (i) a polymer having a selectivity for the aimed gas over the second gas, and (ii) functional groups covalently connected to the polymer as part of the backbone. The functional groups have the capability to further increase the selectivity of the improved polymer via their interaction with the aimed gas and/or with the second gas. All these patented works could find an extended range of industrial utilizations such as wastewater treatment plants, food industries, and oil and gas industries.

### 5.2. Patents on Polymer Nanocomposites as Adsorbent for Environmental Applications

Paul et al. [209] published an invention that reveals sustainable biomaterial scaffold nanocomposites suitable for fluoride removal, reactive black and chromium Cr(VI) removal from wastewater, and a process to synthesize biomaterial scaffold nanocomposites. The authors reported its utilization for the listed contaminants. This biomaterial scaffold nanocomposite exhibited a high fluoride uptake efficiency of 168 mg g^−1^ and 60 mg g^−1^ respectively, at pH 4 and the capacity of the uptake was close to 8.5 mg g^−1^ for Cr(VI) at pH 7. Over 99% reactive black-5 (RB-5) removal was attained with outstanding surface restoration property with this material. Alqadami et al. [210] patented a magnetic polymer nanocomposite for divalent metal ions removal from water. The composite is a magnetic nanocomposite possessing a core of magnetite (Fe_3_O_4_) in a shell of branched polyhydroxystyrene, entitled as Fe_3_O_4_@BHPS. The nanocomposite was prepared via co-precipitation in alkali solution. Results respectively depicted that the nanocomposite attained 93% adsorption of Pb(II) and 80% adsorption of Cd(II), in 30 minutes, reaching equilibrium in 120 minutes. The optimum adsorption capacities of Pb(II) and Cd(II) at 298 K were 186.2 and 125 mg g^−1^, respectively. Finally, the nanocomposite with the heavy metal(s) adsorbed was easily removed from aqueous solution via the utilization of a magnetic field.

As discussed in Section 3.3, information on polymer nanocomposites as adsorbent for separation of gases is minimal, except for other promising adsorbents for the separation of gases. In the same vein, patent work on polymer nanocomposites as adsorbent for separation of gases is minimal; however, there are adsorbents for the separation of gases. Long et al. [211] patented metal-organic frameworks of the family of M_2_ (2,5-dioxido-1,4-benzenedicarboxylate) where some divalent metals are collection of porous crystalline materials made of metal cations or clusters joined by multitopic organic linkers. The metal-organic frameworks can be employed to segregate individual gases from a stream of mixture of gases. This group of adsorbent materials integrates a high density of coordinatively-unsaturated centers lining the pore surfaces. These adsorbents are mainly suitable for selective carbon dioxide/monoxide adsorption through pressure swing adsorption close to temperatures of 313 K. This is because they selectively adsorb carbon dioxide at high pressures in the presence of hydrogen, and desorb CO_2_ when the system experiences a pressure decrease. These materials are also useful for the storage of gases (acetylene storage), and catalysis (oxidation). Allendorf et al. [212] patented a method that includes revealing a gaseous mixture made a noble gas to a metal organic structure, which practically adsorbs a noble gas from a mixture of gases. The noble gas was selectively adsorbed in the adsorbent bed in relation to the other gases in the mixture. Schroder and Yang [213] invented a metal organic structure that consists of a metal ion (M) and an organic ligand having above one hydroxyl ligand. They further provided a method for preparing the metal-organic frameworks and their utilizations in areas such as exhaust gas streams of acidic gases scrubbing, natural gas of acidic gases scrubbing via separation or sequestration, and separating C_2_H_4_ or other volatile organic compounds gases from other gas mixtures. The patented work on adsorbent for environmental applications could also find an extensive range of usage in the industry, like wastewater treatment plants, dye industries, food industries, and oil and gas industries.

On account of the low reactivity of gases springing from their impregnated valence shell, processes that are dependent on sorption, utilizing porous materials are generally problematic. Processes of sorption-based separation must, however, depend mainly on variances in atomic size and weak interactions with surfaces. In addition, there are currently limited publications on polymer nanocomposites as adsorbent for separation of gases. This serves as the motivation for future study.

### 5.3. Future Work on Polymer Nanocomposites for Environmental Applications

In the past decade, studies have shown that polymer nanocomposites for practical applications in membranes and adsorbents for water treatment and gas separation have proven to have distinctive physicochemical properties that cannot be measured when discrete components are used. Nanocomposites are principally multiphase solid materials that include gel, porous media, colloids, and copolymers. The selection of hosts for nanocomposites is of great magnitude because it governs the performance of nanocomposites [214]. It is therefore necessary to research polymer nanocomposites based on the production of tailored materials that possess the ability to control nanocomposite performance in water purification and gas separation. In addition, it is very important to investigate polymer nanocomposite based on sustainability and reuse for high performance in water treatment and gas separation for commercial competence. This will enable its commercial utilizations owing to its ease of use, flexibility, and adaptability.

Furthermore, advancements in polymer nanocomposites have raised concerns with regards to their potential extreme environmental effects in the past decade. Regarding the negative environmental effect, an example of such concern is the difficulty in eliminating graphene from waste. This is a result of the noxious property of nanocomposite graphene and fire outbreak risk of nanocomposite graphene owing to their thermal conductivity and fire retardancy properties. In addition, there are worries regarding unidentified damaging impacts of nanomaterials and the detrimental effects of toxicity of nanomaterials [215]. Hence, adequate methods of evaluating toxicity in polymer nanocomposite is very important for the confirmation of their practical and commercial applications. Besides, the evaluation and contrast of several polymer nanocomposite performance in water treatment are documented standards [216]. It is however, difficult to correlate the performances of these nanoparticles and draw out outstanding nanocomposites that intrinsically worth sustainable application. As a result, researchers should consider putting in place performance assessment tool for water purification.

## 6. Conclusions

The utilization of nanotechnology in different environments has upgraded the present-day environmental engineering and science together with a fresh set of technology that emerged from nanotechnology. The emerged technology at nanoscale has stimulated the advanced utilization of innovative and low-cost techniques that are effective for filtration and adsorption processes for the removal of contaminants in environment. Polymer nanocomposites are materials that make use of advantages of the two materials (nanomaterials and polymers) that makes the composites. The utilization of these materials have resulted in the development of highly efficient materials for environmental utilizations, which have globally gotten the interest of academia and industry. In addition, the progresses in polymer nanocomposite materials properties have allowed numerous industrial utilizations. As an illustration, the integration of engineered nanoscale materials into a polymer membrane matrix has recently earned a substantial consideration for wastewater treatment utilizations.

Different compositions and morphologies of polymer nanocomposites have the capacity of providing great tools for the ecological applications. Furthermore, liberty to functionalize the nanomaterials using several chemical groups could also upsurge their affinity in the direction of target contaminants. This is immensely attractive for selective extraction of target analytes in multifaceted environmental matrices. Hence, main novelties in polymer nanocomposites for environmental utilizations is been reviewed. This review summarizes the different aspects of polymer nanocomposites for environmental applications together with their mechanisms and features of the transport and dissolution of substances in polymer nanocomposites. Patents on the employment of polymer nanocomposites innovations for environmental utilizations were discussed. The review has shown that polymer nanocomposites have an excellent potential for environmental applications as it offers cutting-edge, up-to-date research.

## Figures and Tables

**Table 1:** Exceptional properties of different nanomaterials.

Nanomaterials	Properties	References
Nanoparticles	-Possess huge surface area to volume ratio -Possess high percentage of atoms/molecules associated with surfaces -Have exceptional chemical and physical properties. -Possess unique optical properties that depend on the size, which conveys diverse colours as a result of absorption in the noticeable section. -Possess high reactivity and toughness properties that depend on their distinctive structure, size, and shape. -Possess strong particle mobility -Possess strong surface energy and colloid stabilisation via the provision of barricade to close approach of two particles. -Possess same size scale as many biological molecules.	[2,3,12,13,14,15]
Carbon nanotubes	-Possess high thermal conductivity -Possess a remarkable electrical conductivity -Possess a remarkable mechanical property -Possess a large length-to-diameter ratio (aspect ratio) of higher than 1000 -The images of the actual space examination of nanotube have revealed a series of inter-stratum spacing -Single walled nanotube generally comprises of only 10 atoms near the circumference and the thickness of the tube is only one-atom thick	[3,16,17,18]
Nanosheets	-Exhibits high surface area that makes them advantageous for the fabrication of excellent reinforced polymeric composites -Their surfaces contain a large quantity of active oxygen-containing groups -Possess excellent mechanical and thermal conductivity properties -Possess excellent catalytic activities such as photo-/thermo-catalytic activity -Possess excellent thermal and electrical conductivity	[5,6,19,20,21]
Nanofibers	-Nanofibers are very small in size, which accords them outstanding physical and chemical properties -Possess huge surface area, high aspect ratio, and superior surface properties, which is responsible for their suitability for other technologies that need a smaller environment for chemical reaction to take place -Possess high pore volume and tight pore size that accords their suitability for an extensive range of filtration applications -Possess extreme adsorption capacity that has the capacity to improve many applications	[4,22,23,24]
